# Supplementary material for: How long do floods throughout the millennium remain in the collective memory?
Source: Nat Commun. 2019 Mar 7;10:1105. doi: 10.1038/s41467-019-09102-3 (PMC6405947; doi:10.1038/s41467-019-09102-3)
Supplement: Supplementary file 1 — Supplementary Information [file 41467_2019_9102_MOESM1_ESM.pdf]

Fanta et al.: How long do floods throughout the millennium remain in the collective memory?

* Correspondence to: fanta.vaclav@gmail.com

Supplementary Information

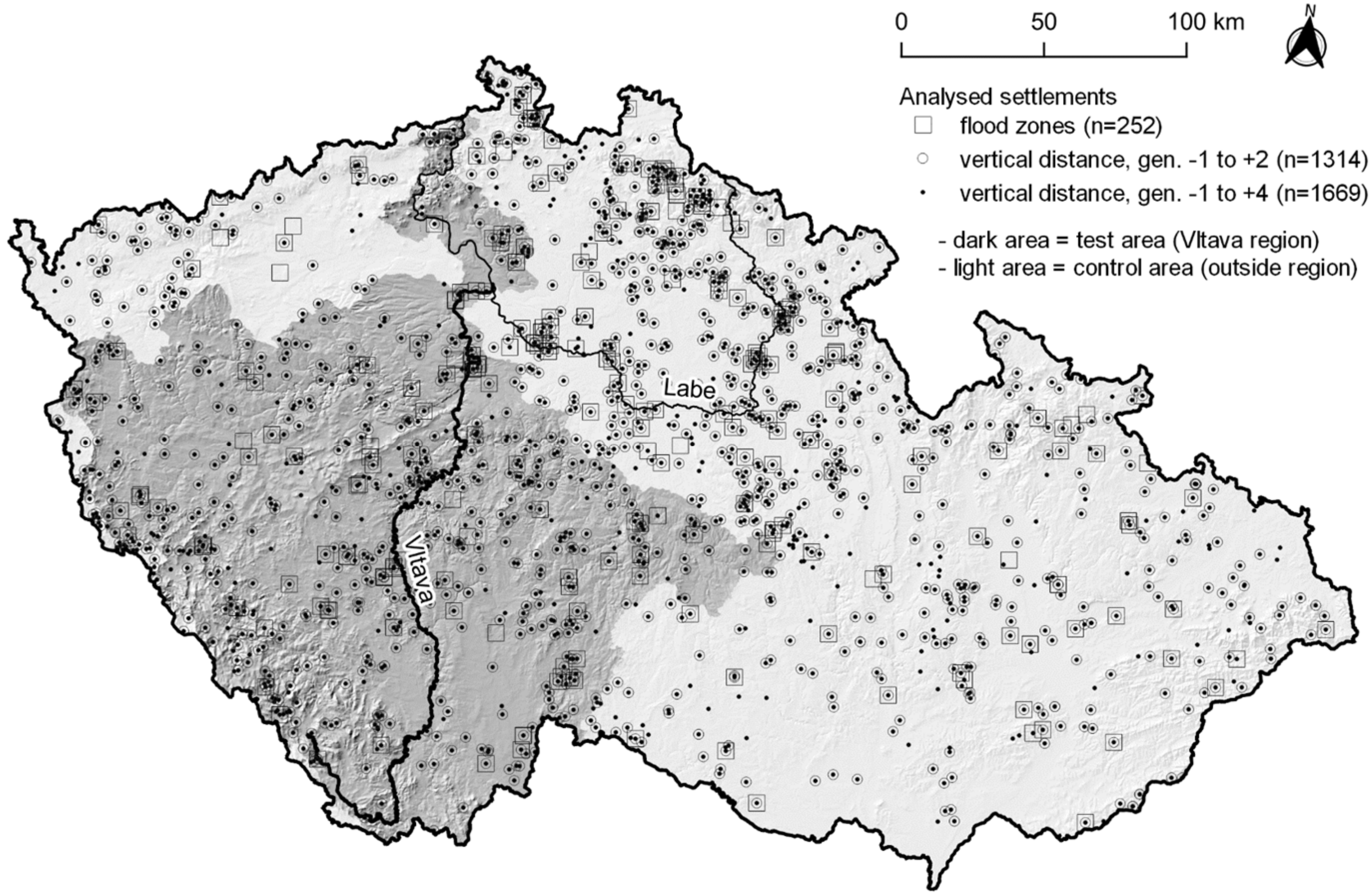

Supplementary Figure 1: Distribution of the analysed settlements within the Czech Republic.

The test area (Vltava region) and the control area (outside region) within the Czech Republic. The border of the test area has been delimited as the Vltava river catchment plus a few smaller drainage areas below the confluence of the Vltava (Moldau) and the Labe (Elbe). The geographical data were downloaded from [1–4]

Supplementary Table 1: Numbers of settlements selected for the analysis.

In one case, the before/after intervals of adjacent floods overlapped each other (the study periods between the floods in 1784 and in 1845 overlapped in years 1820 – 1833). The settlements in the overlap (n = 21) were included in both intervals

Flood no.	Flood [year]	Generation no.	25-year intervals before and after the flood	Historical dating controlled by archaeological dating [No.]		Archaeological dating [No.]		Historical dating after 1600 [No.]		Sum [No.]		Total (generations -1, 1 and 2 only) [No.]	Total [No.]
				test	control	test	control	test	control	test	control		
		-1	1093-1118	2	0	0	2			2	2		
		1	1118-1143	1	4	0	0			1	4	**62**	
**1**	**1118**	2	1143-1168	3	2	28	20			31	22		**87**
		3	1168-1193	3	0	3	1			6	1		
		4	1193-1218	3	4	7	4			10	8		
		-1	1317-1342	5	8	0	2			5	10		
**2**	**1342**	1	1342-1367	5	16	7	9			12	25	**62**	**69**
		2	1367-1392	2	3	4	1			6	4		
		3	1392-1407	4	3	0	0			4	3		
		-1	1407-1432	1	2	0	0			1	2		
**3**	**1432**	1	1432-1457	1	0	4	10			5	10	**25**	**25**
		2	1457-1476	3	2	1	1			4	3		
		-1	1476-1501	1	2	0	0			1	2		
		1	1501-1526	0	2	0	1			0	3	**17**	
**4**	**1501**	2	1526-1551	0	7	3	1			3	8		**34**
		3	1551-1576	1	1	0	1			1	2		
		4	1576-1601	0	1	0	0	6	7	6	8		
		-1	1630-1655	1	0	0	2	92	120	93	122		
		1	1655-1680	0	0	0	0	12	51	12	51	**363**	
**5**	**1655**	2	1680-1705	0	0	0	0	20	65	20	65		**658**
		3	1705-1730	0	0	0	0	70	122	70	122		
		4	1730-1755	0	0	0	0	30	73	30	73		
		-1	1759-1784	0	0	0	0	22	89	22	89		
**6**	**1784**	1	1784-1809	1	0	0	0	174	276	175	276	**591**	**602**
		2	1809-1834	0	0	0	0	6	23	6	23		
		3	1834-1845	0	0	0	0	1	10	1	10		
		-1	1820-1845	0	0	0	0	77	60	77	60		
**7**	**1845**	1	1845-1870	1	0	0	0	24	26	25	26	**194**	**194**
		2	1870-1895	0	0	0	0	4	2	4	2		
			**Sum [No.]**	40	58	58	56	538	924	636	1038		
			**Total [No.]**	**98**		**114**		**1462**				**1314**	**1669**

**Generation -1 [No.]**	492
**Generation 1 [No.]**	626
**Generation 2 [No.]**	201
**Generation 3 [No.]**	220
**Generation 4 [No.]**	135

Supplementary References

1. Arcdata Praha. ArcČR® 500, version 3.3 [ESRI database]. (2016). Available at: http://download.arcdata.cz/data/ArcCR500-3.3-windows-installer.exe. (Accessed: 12th March 2017)

2. Land Survey Office. WMS view service – DMR 5G (Hill Shaded Terrain Model) [WMS data]. *Geoportál ČÚZK* (2017). Available at: http://geoportal.cuzk.cz/(S(lvw4nmwqqcz1ycdyxtbrrun3))/Default.aspx?menu=3130&mode=TextMeta&side=wms.verejne&metadataID=CZ-CUZK-WMS-DMR5G&metadataXSL=metadata.sluzba. (Accessed: 15th June 2017)

3. T. G. Masaryk Water Research Institute. Hydrological classification [data]. *DIBAVOD [Digital Database of Water Management Data]* (2017). Available at: http://www.dibavod.cz/download.php?id_souboru=1421&PHPSESSID=3eed5341964ceb85a88e8e54b32f0d61, http://www.dibavod.cz/download.php?id_souboru=1420&PHPSESSID=3eed5341964ceb85a88e8e54b32f0d61, http://www.dibavod.cz/download.php?id_souboru=1419&PHPSESSID=3eed. (Accessed: 3rd April 2017)

4. T. G. Masaryk Water Research Institute. Watercourses [data]. *HEIS VÚV TGM [TGM WRI Hydroecological Information System]* (2012). Available at: http://heis.vuv.cz/data/webmap/datovesady/HEIS/UtvaryPOV/E_HEIS$UPOV_R.zip. (Accessed: 24th January 2015)
