# Supplementary material for: How long do floods throughout the millennium remain in the collective memory?
Source: Nat Commun. 2019 Mar 7;10:1105. doi: 10.1038/s41467-019-09102-3 (PMC6405947; doi:10.1038/s41467-019-09102-3)
Supplement: Supplementary file 3 — Description of Additional Supplementary Files [file 41467_2019_9102_MOESM3_ESM.pdf]

Description of Additional Supplementary Information

File Name: Supplementary Data 1
Description: Vertical distance of real settlements (all towns and villages)

File Name: Supplementary Data 2
Description: Vertical distance of real settlements (25 years before flood to 50 years after flood)

File Name: Supplementary Data 3
Description: Vertical distance of virtual settlements

File Name: Supplementary Data 4
Description: Presence in flood zones - real settlements (50 years before flood to 50 years after flood)

File Name: Supplementary Data 5
Description: Presence in flood zones - virtual settlements
